# Mental, neurological and substance use disorders among the Latino migrant population in the United States who visited the Health Windows and Mobile Health Units in 2021

**DOI:** 10.3389/fpubh.2023.959535

**Published:** 2023-02-06

**Authors:** Ana María López Jaramillo, María Gudelia Rangel Gómez, Silvia Morales Chainé, Alejandra López Montoya, Isaura Angélica Lira Chávez, Rodolfo Cruz-Piñeiro

**Affiliations:** ^1^El Colegio de la Frontera Norte, Tijuana, Mexico; ^2^Comisión de Salud Fronteriza México—Estados Unidos, Tijuana, Mexico; ^3^Universidad Nacional Autónoma de México, Mexico City, Mexico

**Keywords:** mental health, migrant population, Health Windows, Mobile Health Units, substance use disorders (SUD)

## Abstract

**Background:**

Mental health is defined by the World Health Organization as a state of wellbeing in which people are aware of their own abilities to cope with the normal stresses of life, work productively and fruitfully, and contribute to their community. Among the minority groups that may be vulnerable to experiencing greater risks for their physical and mental health and full development is the migrant population. The mobile population's migration experience, from their place of origin to destination translates into psychosocial problems and clearly stressful conditions which could be resolved using certain coping strategies. Accordingly, numerous epidemiological studies have found differences in the prevalence of mental health problems between migrants and native-born residents of destination countries, as well as between migrants and their non-migrant co-nationals.

**Purpose:**

To describe sociodemographic characteristics of the Latino migrant population in the United States who visited the Health Windows (HW) and Mobile Health Units (MHU) in 2021, who may have been at risk for mental, neurological or substance use disorders and agreed to a screening for signs and symptoms of mental health conditions.

**Method:**

Users of the HW and MHU were offered preventive health services and completed a mental health screening. These variables were registered in SICRESAL. If their results showed signs and symptoms of mental health conditions, they were screened by credentialed professionals from the Psychology Faculty of the National Autonomous University of Mexico. Screened individuals received a diagnosis and specialized care remotely and/or online with the MHU and HW network partners. To analyze sociodemographic variables corresponding to neurological or substance induced mental illness among the Latino migrant population in the United States who visited the Ventanillas de Salud (VDS)/Health Windows (HW), and Unidades Móviles de Salud (UMS)/Mobile Health Units (MHU), during 2021; contingency tables were created showing percentages and chi square with a significant *p* < 0.05.

**Findings:**

During 2021 HW and MHU completed a total of 794 mental health screenings of which 84% were completed at HW. Further, 59% were women with an average age of 43, ranging from 7 to 86 years of age. Twenty percent 20% of the population who voluntarily agreed to screening yielded a positive result for some type of mental health symptom or problem. This percentage (37%) was greater among those who consulted MHU. With respect to age, results showed that youth were at greatest risk for mental health problems. Among the screened population, the independent variables, type of Health Window attended, gender, age group, and place of origin are related to the existence of some type of mental health symptom or problem yielding a significance level of <0.05 for depression and anxiety symptoms.

**Discussion and prospects:**

In this study, as in others, the migrant population that visited the HW and UMS in 2021 reported a greater risk of mental health problems, with symptoms related to depression and anxiety among the socio-demographic variables of gender, age group, and place of origin. Thus, these symptoms relate to being a female aged between 18 and 38 and originating from Mexico. Finally, the possibility of screening the migrant population for signs and symptoms of mental health conditions that attended the Health Windows or Mobile Health Units during 2021, made it possible to refer them to psychology or psychiatry services and improve the quality of life of those who accessed the services and, consequently, that of their families and communities.

**Limitations:**

The main limitation is associated with the information source since we worked with secondary data and relied on the information provided by those who attended both the HW and the MHU.

## 1. Mental health: Concepts and scope

### 1.1. Defining mental health: World status and Mexico

Mental health is defined by the World Health Organization (1) as a state of wellbeing in which people are aware of their own abilities to cope with the normal stresses of life, work productively and fruitfully, and contribute to their community.

Causes of mental disorders include numerous factors or determinants such as individual or biological characteristics (genetic or environmental and biological) defined as the ability to manage thoughts, emotions, and interactions with others (2). They also comprise psychosocial factors, which are related to the physical and social environment. This encompasses social, cultural, economic, political, and environmental aspects such as the political context, including national policies, welfare, living standards, working conditions and community support networks (2).

According to the mhGAP intervention guide for mental, neurological and substance use disorders in non-specialized health settings: mental health Gap Action Programme (3), the main clinical manifestations of mental illness reflect an alteration of brain functions, such as attention (attention deficit disorder), memory (dementia), thinking (schizophrenia), mood (depression), sensory perception (schizophrenia), learning (child development disorders) and behavior, which interfere with the life and productivity of the individual.

As Morales (4) notes, various social phenomena such as poverty, urban violence, family violence, intense pressure in the workplace, job insecurity, low social support, addictive behaviors, family disintegration, street children, sexual exploitation, and the physical abuse of children, are considered factors that are associated with or influence the mental health of a population.

The “Report on mental health systems in Latin America and the Caribbean, 2013,” published by the Pan American Health Organization (5), notes that lifetime prevalence rates of between 12.2% and 48.6% have been estimated for mental disorders worldwide. Likewise, 14% of the global disability burden (Disability-Adjusted Life Years—DALYs) is attributable to mental health conditions and the situation is even more critical in low- and middle-income countries. In these countries, between 76% and 85% of those suffering from serious mental illnesses or disorders fail to receive treatment. In high-income countries, these proportions range from 35% to 50% (5).

In Mexico, the “Report on the mental health system in Mexico, 2011,” undertaken by the WHO, PAHO and the Ministry of Health (6), observes that one in four Mexicans aged between 18 and 65 has suffered a mental disorder at some point in their lives. However, only one in five received treatment.

There are minority groups such as Indigenous people, people subjected to discrimination and rights violations, migrants, the LGTBI population, prisoners, those exposed to armed conflicts or natural disasters or other humanitarian emergencies who experience social vulnerability that may place them at increased risk for MH issues. This risk is a condition experienced by people who, due to their age, gender, ethnic origin or physical limitations, experience greater threats to their health, physical and mental integrity and full development, and whose membership of certain groups makes them vulnerable (4).

Specifically, the migration experience is one such major stressor that can increase social vulnerability. The upheavals and stressful conditions associated with migration translate into psychosocial problems throughout life (7). This is why it is intended to describe the sociodemographic profile of the Latino migrant population in the United States who visited the Health Windows (HW) and Mobile Health Units (MHU) in 2021, who may have been at risk for mental, neurological or substance use disorders and agreed to undergo screening.

### 1.2. Mental health and migration

The association between migration and mental health problems has been documented for many years (8, 9). Some of the earliest studies on the relationship between migration and mental health include those by Ødegaard (10), undertaken in the 1930s. This author observed that Norwegian immigrants in the United States had a higher incidence of hospital admission for schizophrenia, compared to both native-born residents of that country and non-migrant Norwegians. Bojórquez (11) notes that this and other similar findings sparked a discussion on the two possible forms of association between migration and mental health: causality or selection. According to the former position, migration is a risk or precipitating factor for the emergence of mental health problems. According to the latter, it is a self-selection phenomenon, whereby people with mental health problems are more likely to emigrate.

Authors such as Vilar and Eibenschutz (12) emphasize that perhaps migration is not a direct cause of deterioration of mental health *per se*; more so, can be attributed to relocation from their place of origin to a new place of destination, in which they experience adverse employment situation, poor housing conditions or lack of housing, the traumatic events before, during and after the migration. These factors could be considered sufficient reasons to drive individuals to psychological distress.

Further, Achotegui (13) noted that migration brings about benefits (access to new vital opportunities and horizons) paired with a group of difficulties and strains. Thus, a migrant's lack of health or disabilities could become a risk factor given a hostile environment or experiencing both conditions at their point of destination.

Several epidemiological studies have found differences in the prevalence of mental health risks between immigrants and native-born residents of destination countries, as well as between immigrants and their non-migrant co-nationals. Explanations for the higher prevalence of mental disorders among immigrants than non-immigrants include the fact that migration is a stressful life experience, in which bereavement combines with the difficulties of adapting to a new culture and, in many cases, the risk of discrimination and violence (9, 14, 15).

Mexican-born immigrants have the lowest rates of mental illness, which increase with the length of time spent in the United States and generational descent. The second generation of Mexicans in the United States have higher rates of mental disorders than those observed in the general population.

A smaller proportion, depending on their mental health history, violence experienced before, during and after their journey and personal resources, will present mental disorders (depression, anxiety, post-traumatic stress, and substance abuse disorders) and require specialized services (16).

The mental conditions most frequently reported and described are those related to experiences of psychosis and paranoid reactions with a tendency to affective disorders, unipolar depression, anxiety, adaptation difficulties, alcohol dependence and post-traumatic stress (17).

Another important aspect with mental health is the abuse of other substances such as psychoactive substances. Some studies report that consumption increases in tandem with greater exposure to North American culture (18). These results are related to the returned population, with 28% of Mexican migrants who returned voluntarily or forcibly from the United States self-reporting lifetime illegal drug use. A third of female returning migrants between the ages of 15 and 45 have used drugs at some time, which is higher than the rate for women of the same age in Mexico.

Regarding the deported migrant population, a study undertaken by Bojórquez et al. (19) notes that the length of time after having returned to Mexico, having a spouse in the US, the number of household members, less social support, anxiety, and an avoidant coping style were directly associated with the self-report questionnaire score. Public health policies must address the need for mental health care among deported migrants (19).

In view of this situation, a collaboration strategy was developed between the Government of Mexico and the Faculty of Psychology of the National Autonomous University of Mexico, through which training, supervision and two-way monitoring are undertaken by the non-specialized health personnel who provide services to the migrant population at the Health Windows (HW) and Mobile Health Units (MHU). As a result of the collaboration protocol, it was possible to undertake promotion and early care actions for mental health among the migrant population that visited these Windows or Units.

As Rangel et al. (20) notes, the mission of HW is to improve access to primary and preventive health services, increase public health insurance coverage, link individuals to medical homes, and promote a culture of self-care among Mexicans living in the US. In addition to general health information, the HW provides (a) counseling and guidance services to prevent risks to physical and mental health; (b) screening for mental health risks; (c) referrals for those with mental health risks to primary care services; and (d) information about the eligibility for the health insurance plans of the Patient Protection and Affordable Care Act (ACA).

MHU also provide health education on priority health topics such as nutrition, obesity, diabetes, women's health, children's health, mental health, substance use, exposure to violence, HIV/AIDS, and other sexually transmitted diseases, as well as legal and financial guidance. In addition, Mobile Health Units provide preventive health screenings, referrals to clinics or community programs, follow up referrals, and administer immunizations (20).

This paper seeks to describe the sociodemographic characteristics of the Latino migrant population in the United States who visited the Health Windows (HW) and Mobile Health Units (MHU) in 2021, who may have been at risk for mental, neurological or substance use disorders. The database of the Continuous Information System for Health Reporting (SICRESAL), developed and maintained by the Mexico Section of The United States-Mexico Border Health Commission, was used as the information source. Data collected from the HW and MHU, based on the basic sociodemographic characteristics, signs and symptoms of mental health diseases yielded by the screening of the population served, was reviewed and analyzed.

## 2. Methodology

### 2.1. Design

The collaboration strategy, through training, supervision and bidirectional monitoring among non-specialized health personnel who care for the migrant population at the HW and MHU, allowed for promotion and early care for mental health.

This strategy included the following activities:

1. 40-h training for non-specialized health personnel who care for the migrant population at HW and MHU, on prevention and early care for mental health problems.
2. Coordination of the strategy for the implementation of brief mental health interventions for compatriots cared for at the community level, initially, by VDS and UMS promoters and, subsequently, referred to primary and specialized care, by professionals from the Faculty of Psychology of the UNAM.
3. Coordinate the monitoring and supervision of 37 VDS and UMS promoters who implemented the evaluation, management and follow-up of mental health risk conditions, throughout 11 synchronous sessions, between April and December 2021: essential care/practices depression, psychosis, epilepsy, child/adolescent mental/behavioral disorders, dementia, substance use disorders, self-harm/suicide, and other conditions such as acute stress and violence.
4. Evaluate the implementation process of mental health interventions through the development of three evaluation processes: (a) knowledge, (b) case vignettes, and (c) simulated situations of people with risk to their mental health.

The mental health initiative, called the “Mental Health Gap Action Program (mhGAP),” involved (1) training promoters; (2) evaluation, monitoring, and follow-up; and (3) mental health screening for HW and MHU users. In other words, those treated at HW and MHU were provided with preventive and screening services for mental health risks, with the results being recorded in SICRESAL.

If the promoters of the HW and MHU network partners identified mental health conditions, individuals were then screened by, diagnosed and offered specialized care remotely and/or online from the Faculty of Psychology of the National Autonomous University of Mexico.

It should be noted participation was voluntary. Through direct work in VDS and UMS health promoters gently encouraged participants to accept receiving screening as well as remote mental health services, if necessary.

### 2.2. Information source

De-identified data from the *Continuous Information System for Health Reporting (SICRESAL)* housed by the Mexico Section of the United States-Mexico Border Health Commission were used for the analysis. In 2021, a total of 36,086 Mexicans received community health care at the HW and 10,384 in the MHU. A total of eighty-three were screened by the HW (71% women, with an average age of forty-two, between the ages of 14 and 67), and 127 by the MHU (69% women, with an average age of forty-one, between the ages of 19 and 73).

### 2.3. Instruments

The variables included in the screening are associated with the sociodemographic profile such as age, sex, state of residence and medical insurance, as well as mental health signs and symptoms related to depression (six items), experiences of psychosis (four items), epilepsy (one item), dementia (two items), substance use (three items), violence (four items), sexuality (two items), anxiety (three items) self-harm and suicidal ideation (two items). One point was assigned to each item and the occurrence of the event was determined as follows: (a) For depression, anxiety (in the past 2 weeks) and dementia (at the time of assessment), with at least two positive items; and (b) for the experience of psychosis, epilepsy, sexuality (in the past 12 months), violence (in the past 6 months), substance use (in the past month), self-harm and suicidal ideation (at the time of administration) with at least one positive item.

### 2.4. Data analysis

It is important to clarify that no sample calculation was made for this study since it is an exploratory study that aimed to describe the sociodemographic characteristics of the Latino migrant population that voluntarily agreed to the mental health screening offered at both the HW and MHU.

Based on the information contained in the SICRESAL database, the sociodemographic characteristics of those served in both the HW and the MHU in 2021 was constructed, analyzing variables such as gender, age groups, English proficiency, place of origin and place where there were attended.

In the analysis of sociodemographic variables relating to mental illness, neurological or due to substance use, among the Latino migrant population in the United States attending HW and MHU during 2021, contingency tables were calculated with respective percentages and chi square score with a significant *p* < 0.05. The initial part of the analysis included “any mental health symptom or problem” as a dependent variable; the sociodemographic dependent variables were gender, age group, place of origin, and English language proficiency. The demographic variables of education and time of residency in the USA were not analyzed given the lack of information among registrants. In the second part, used SPSS to analyze the information using the dependent variables, symptoms of depression, anxiety, suicidal ideation, psychosis, epilepsy, dementia, substance use, violence or problems with sexuality, and socio-demographic as explanatory variables.

## 3. Results

### 3.1. Positive mental health screenings data analysis

During 2021, a total of 794 mental health screenings were conducted by the HW and the MHU, 84% were done in the HW. Fifty-nine percent of screenings were done among women between 7 and 86 years of age, with an average age of 43.

Table 1 shows data for population screening with or without mental health symptoms/problems and other socio-demographic variables. The following findings emerged:

- 20% of the population that voluntarily agreed to screening showed positive results for a mental health symptom or problem. This percentage is higher among those who attended the MHU (37%).
- Analyzing for the variable of gender revealed that the aforementioned symptoms were higher among women.
- Those between 18 and 30 years of age were more likely to report having a mental health symptom or problem.
- For 26.9% of the screened population, Mexico was the country of origin, and 26.1% from Central America.
- The independent variables “type of window,” “gender,” “age group,” and “place of origin” were related with the existence of some mental health symptom or problem among the population who agreed to the screening given that its significance level was below 0.05. The variable for language proficiency was neither significant nor explicative.

**Table 1:** Distribution of sociodemographic characteristics among the population with or without mental health symptoms/problems for screenings done at HW and MHU during 2021.

**Characteristics**	**Any symptoms or mental health**				
	**problems**				
	**Yes**		**No**		**Sig**.
***N***	**160**		**634**		
**Health window type**	***n***	**%**	***n***	**%**	
VDS	113	16.9	554	83.1	0.000^*^
UMS	47	37	80	63	
**Gender**					
Women	113	24.1	355	75.9	0.001^*^
Men	47	14.4	279	85.6	
**Age groups**					
5–9 years	0	0	1	100	0.030^*^
10–17 years	2	25	6	75	
18–30 years	31	31.6	67	68.4	
31–40 years	43	15.7	231	84.3	
41–50 years	47	20.3	185	79.7	
51–60 years	33	22.6	113	77.4	
Older than 61 years	4	11.4	31	88.6	
**Place of origin**					
Caribbean	0	0	12	100	0.000^*^
Central America	6	26.1	17	73.9	
United States	3	16.7	15	83.3	
Mexico	141	26.9	383	73.1	
South America	5	20	20	80	
**English proficiency**					
Yes	32	23.4	105	76.6	0.306
No	58	21.6	210	78.4	

* Differences estimated using Chi-square value p < 0.05.

Author's own creation based on Data Base Continuous Information System for Health Reporting (SICRESAL), 2021 (21).

A desegregation of screened mental health symptoms found differing results among the migrant population that attended a HW or MHU during 2021. These are described as follows and shown on Tables 2–**4**.

**Table 2:** Distribution of sociodemographic characteristics among the population with or without mental health symptoms/problems of depression, anxiety, suicidal ideation for screenings done at HW and MHU during 2021.

**Characteristics**	**Symptoms of depression**					**Anxiety symptoms**					**Symptoms of suicidal ideation**				
	**Yes**		**No**		**Sig**.	**Yes**		**No**		**Sig**.	**Yes**		**No**		**Sig**.
***N***	**148**		**646**			**58**		**736**			**10**		**784**		
**Health window type**	***n***	**%**	***n***	**%**		***n***	**%**	***n***	**%**		***n***	**%**	***n***	**%**	
VDS	109	16.3	558	83.7	0.000^*^	45	6.7	622	93.3	0.166	10	1.5	657	98.5	0.165
UMS	39	30.7	88	69.3		13	10.2	114	89.8		0	0	127	100	
**Gender**															
Women	108	23.1	360	76.9	0.000^*^	41	8.8	427	91.2	0.059	6	1.3	462	98.7	0.945
Men	40	12.3	286	87.7		17	5.2	309	94.8		4	1.2	322	98.8	
**Age groups**															
5–9 years	0	0	1	100	0.040^*^	0	0	1	100	0.177	0	0	1	100	0.906
10–17 years	2	25	6	75		0	0	8	100		0	0	8	100	
18–30 years	29	29.6	69	70.4		13	13.3	85	86.7		2	2	96	98	
31–40 years	41	15	233	85		20	7.3	254	92.7		3	1.1	271	98.9	
41–50 years	40	17.2	192	82.8		16	6.9	216	93.1		2	0.9	230	99.1	
51–60 years	32	21.9	114	78.1		9	6.2	137	93.8		3	2.1	143	97.9	
Older than 61 years	4	11.4	31	88.6		0	0	35	100		0	0	35	100	
**Place of origin**															
Caribbean	0	0	12	100	0.000^*^	0	0	12	100	0.005^*^	0	0	12	100	0.390
Central America	6	26.1	17	73.9		2	8.7	21	91.3		0	0	23	100	
United States	2	11.1	16	88.9		1	5.6	17	94.4		0	0	18	100	
Mexico	131	25	393	75		50	9.5	474	90.5		10	1.9	514	98.1	
South America	4	16	21	84		3	12	22	88		0	0	25	100	
**English proficiency**															
Yes	29	21.2	108	78.8	0.467	7	5.1	130	94.9	0.554	3	2.2	134	97.8	0.071
No	53	19.8	215	80.2		21	7.8	247	92.2		0	0	268	100	

* Differences estimated using Chi-square value p < 0.05.

Author's own creation based on Data Base Continuous Information System for Health Reporting (SICRESAL), 2021 (21).

### 3.2. Data analysis of positive mental health screenings for symptoms related to emotional state

Table 2 shows data for population screening with or without mental health symptoms/problems of depression. The following findings emerged:

- 19% of the screened population showed positive results for depression. This percentage is higher among those who attended an MHU (31%).
- The aforementioned symptoms were found to be higher among women (23%).
- The population between 18 and 30 years of age were more likely to report symptoms of depression.
- 26% of the screened population reported Central America as their place of origin.
- The independent variables, “type of window,” “gender,” “age group,” and “place of origin” were related to a symptom of depression among those who agreed to the screening given its significance level was below 0.05. While the variable of English proficiency was neither significant nor explicative.

Table 2 shows data for population screening with or without mental health symptoms/problems of anxiety. The following findings emerged:

- 7% of the population who voluntarily agreed to the screening showed positive results for anxiety. This percentage is higher among those who attended the MHU (31%).
- While analyzing for gender, women showed the greatest proportion of symptoms.
- The population between 18 and 30 years of age reported the most having symptoms related to anxiety.
- Most reported South America as their place of origin.
- Only the variable, “place of origin” was related to showing a symptom of anxiety among the population who agreed to this screening given its significance level was below 0.05. The variables “type of window,” “gender,” “age group,” and “English proficiency” among this population were neither significant nor explicative.

Table 2 shows data for population screening with or without mental health symptoms/problems of suicidal ideation. The following findings emerged:

- Only 1% of the population who voluntarily agreed to the screening showed positive results in terms of suicidal ideation.
- Analyzing for gender found the aforementioned had a greater occurrence among women.
- The population between 18 and 30 years of age reported the most symptoms related to suicidal ideation.
- Most reported Mexico as their place of origin.
- None of the independent variables related to a symptom of suicidal ideation among the population agreeing to the screening given its significant level was above 0.05.

### 3.3. Analysis of data from positive mental health screenings for psychosis, epilepsy, and dementia

Table 3 shows data for population screening with or without mental health symptoms/problems of psychosis. The following findings emerged:

- From the population who voluntarily agreed to screening, 8% had positive results for psychosis. This percentage was higher among those who attended the MHU.
- Analyzing for gender found the aforementioned had a greater occurrence among women.
- The population between 18 and 30 years of age reported the most symptoms related to psychosis.
- 26% of the screened population reported South America as their place of origin.
- The independent variables “place of origin” and “English proficiency” were the only variables related to the presence of any symptom of psychosis among the population who agreed to the screening given its significance level was below 0.05. The variables “type of window,” “gender,” and “age groups” among this population were neither significant nor explicative.

**Table 3:** Distribution of sociodemographic characteristics among the population with or without mental health symptoms/problems of psychosis, epilepsy, or dementia for screenings done at HW and MHU during 2021.

**Characteristics**	**Symptoms of psychosis**					**Epilepsy symptoms**					**Dementia symptoms**				
	**Yes**		**No**		**Sig**.	**Yes**		**No**		**Sig**.	**Yes**		**No**		**Sig**.
***N***	**64**		**730**			**20**		**774**			**12**		**782**		
**Health window type**	***n***	**%**	***n***	**%**		***n***	**%**	***n***	**%**		***n***	**%**	***n***	**%**	
VDS	52	7.8	615	92.2	0.531	15	2.2	652	97.8	0.266	11	1.6	656	98.4	0.466
UMS	12	9.4	115	90.6		5	3.9	122	96.1		1	0.8	126	99.2	
**Gender**															
Women	43	9.2	425	90.8	0.162	9	1.9	459	98.1	0.199	6	1.3	462	98.7	0.526
Men	21	6.4	305	93.6		11	3.4	315	96.6		6	1.8	320	98.2	
**Age groups**															
5–9 years	0	0	1	100	0.567	0	0	1	100	0.288	0	0	1	100	0.665
10–17 years	0	0	8	100		0	0	8	100		0	0	8	100	
18–30 years	11	11.2	87	88.8		0	0	98	100		1	1	97	99	
31–40 years	26	9.5	248	90.5		12	4.4	262	95.6		2	0.7	272	99.3	
41–50 years	13	5.6	219	94.4		4	1.7	228	98.3		5	2.2	227	97.8	
51–60 years	11	7.5	135	92.5		3	2.1	143	97.9		4	2.7	142	97.3	
Older than 61 years	3	8.6	32	91.4		1	2.9	34	97.1		0	0	35	100	
**Place of origin**															
Caribbean	0	0	12	100	0.001^*^	0	0	12	100	0.170	0	0	12	100	0.280
Central America	1	4.3	22	95.7		0	0	23	100		0	0	23	100	
United States	1	5.6	17	94.4		0	0	18	100		0	0	18	100	
Mexico	57	10.9	467	89.1		19	3.6	505	96.4		12	2.3	512	97.7	
South America	3	12	22	88		0	0	25	100		0	0	25	100	
**English proficiency**															
Yes	8	5.8	129	94.2	0.021^*^	6	4.4	131	95.6	0.292	3	2.2	134	97.8	0.704
No	14	5.2	254	94.8		5	1.9	263	98.1		3	1.1	265	98.9	

* Differences estimated using Chi-square value p < 0.05.

Author's own creation based on Data Base Continuous Information System for Health Reporting (SICRESAL), 2021 (21).

Table 3 shows data for population screening with or without mental health symptoms/problems of epilepsy. The following findings emerged:

- 3% of the population who voluntarily accepted the screening showed positive results for epilepsy. This result is higher among those who attended the MHU.
- While analyzing the variable of gender the aforementioned symptoms showed a major proportion among men.
- The population older than 60 years of age reported the most having symptoms related to epilepsy.
- Most reported Mexico as their place of origin.
- The majority reported English proficiency.
- None of the independent variables was related to a symptom of epilepsy among the population who agreed to the screening given its significant level was above 0.05.

Table 3 shows data for population screening with or without mental health symptoms/problems of dementia. The following findings emerged:

- Only 1% of the population who voluntarily agreed to the screening had positive results for dementia.
- The aforementioned symptoms were present mostly among men.
- The population between 51 and 60 years of age reported the most having symptoms related to dementia.
- Most reported Mexico as their place of origin.
- None of the independent variables was related to the existence of some symptom of dementia among the population who agreed to the screening given its significant level was above 0.05.

### 3.4. Analysis of data from positive mental health screenings for symptoms related to behavior

Table 4 shows data for population screening with or without mental health symptoms/problems with substance use. The following findings emerged:

- 5% of the population who voluntarily agreed to the screening had positive results for substance use.
- The percentage is higher among those who attended the MHU.
- Analyzing for the gender variable showed the aforementioned symptoms were greater among men.
- The population between 41 and 50 years of age reported the most having symptoms related to substance use.
- The majority reported Central America as its place of origin.
- Only the independent variables “type of window,” “gender,” and “place of origin” were related to the existence of some type of symptom related with substance use among the population who agreed to the screening given its significance level was below 0.05. The variables “age group” and “language proficiency” among this population were neither significant nor explicative.

**Table 4:** Distribution of sociodemographic characteristics among the population with or without mental health symptoms/problems of substance consumption, violence or sexuality for screenings done at HW and MHU during 2021.

**Characteristics**	**Symptoms of substance use**					**Symptoms of violence**					**Symptoms of sexuality**				
	**Yes**		**No**		**Sig**.	**Yes**		**No**		**Sig**.	**Yes**		**No**		**Sig**.
***N***	**39**		**755**			**35**		**759**			**35**		**759**		
**Health window type**	***n***	**%**	***n***	**%**		***n***	**%**	***n***	**%**		***n***	**%**	***N***	**%**	
VDS	24	3.6	643	96.4	0.000^*^	33	4.9	634	95.1	0.090	30	4.5	637	95.5	0.778
UMS	15	11.8	112	88.2		2	1.6	125	98.4		5	3.9	122	96.1	
**Gender**															
Women	17	3.6	451	96.4	0.046^*^	23	4.9	445	95.1	0.405	22	4.7	446	95.3	0.630
Men	22	6.7	304	93.3		12	3.7	314	96.3		13	4	313	96	
**Age groups**															
5–9 years	0	0	1	100	0.545	0	0	1	100	0.952	0	0	1	100	0.873
10–17 years	0	0	8	100		0	0	8	100		0	0	8	100	
18–30 years	9	9.2	89	90.8		5	5.1	93	94.9		4	4.1	94	95.9	
31–40 years	12	4.4	262	95.6		14	5.1	260	94.9		16	5.8	258	94.2	
41–50 years	11	4.7	221	95.3		8	3.4	224	96.6		9	3.9	223	96.1	
51–60 years	6	4.1	140	95.9		7	4.8	139	95.2		5	3.4	141	96.6	
Older than 61 years	1	2.9	34	97.1		1	2.9	34	97.1		1	2.9	34	97.1	
**Place of origin**															
Caribbean	0	0	12	100	0.033^*^	0	0	12	100	0.017^*^	0	0	12	100	0.037^*^
Central America	2	8.7	21	91.3		0	0	23	100		1	4.3	22	95.7	
United States	1	5.6	17	94.4		0	0	18	100		0	0	18	100	
Mexico	34	6.5	490	93.5		33	6.3	491	93.7		31	5.9	493	94.1	
South America	1	4	24	96		1	4	24	96		2	8	23	92	
**English proficiency**															
Yes	10	7.3	127	92.7	0.291	6	4.4	131	95.6	0.175	6	4.4	131	95.6	0.079
No	10	3.7	258	96.3		7	2.6	261	97.4		6	2.2	262	97.8	

* Differences estimated using Chi-square value p < 0.05.

Author's own creation based on Data Base Continuous Information System for Health Reporting (SICRESAL), 2021 (21).

Table 4 shows data for population screening with or without mental health symptoms/problems with violence. The following findings emerged:

- 4% of the population who voluntarily agreed to the screening showed positive results for violence. This percentage is higher among those who attended the HW.
- Analyzing gender showed the aforementioned symptoms were greater among women.
- The population between 31 and 40 years of age reported the most having symptoms related to violence.
- Most reported Mexico as their place of origin.
- The majority reported English proficiency.
- Only the independent variable “place of origin” was related to the existence of some symptom among the population who voluntarily agreed to the screening given its significant level was below 0.05. The other independent variables were not related to the existence of a symptom of violence among the population who agreed to the screening given its significance level was above 0.05.

Table 4 shows data for population screening with or without mental health symptoms/problems with sexuality, the following findings emerged:

- Only 4% of the population who voluntarily agreed to the screening showed positive results for sexuality.
- Analyzing for the gender variable found the aforementioned symptoms occurred mostly among women.
- The population between 31 and 40 years of age reported the most having symptoms related to dementia.
- The majority reported South America as its place of origin.
- Only the independent variable “place of origin” was related to the existence of a symptom among the population who voluntarily agreed to the screening given that its significance level was below 0.05. The remaining variables were not related to the existence of any symptom of violence among the population who agreed to the screening given its significance level was above 0.05.

## 4. Conclusions and discussion

Migration is an experience in which more and more individuals participate, and which has potentially negative as well as positive consequences for health and wellbeing (22). This can also be considered a psychic or mental risk factor thus it is necessary to address from a health and psychosocial perspective (23). The main purpose of this study was to describe the sociodemographic characteristics of the Latino migrant population in the United States of America who visited the Health Windows (HW) and Mobile Health Units (MHU) during 2021 and who may have been at risk for mental, neurological or substance use disorders.

The study results allow one to gain a deeper understanding of the socio-demographic portrait of the migrant population attending MHU and HW showing a symptom or problem related with mental health in order to adapt needed care and therapeutic interventions. The significant variables in this analysis were gender, age group, and place of origin.

As reported by other studies on mental health and migration, there is a high prevalence of disorders among the migrant population (13). In this study, the proportion of the population with mental health symptoms or problems identified through the screening was 20%. Specifically, 17% in the HW and 37% in the MHU.

In 2021, the mental health screening undertaken in both the HW and the MHU revealed conditions of stress, anxiety, major depression, psychological distress, risks of self-harm/suicide, psychoactive substance use and post-traumatic stress among the migrant population that went to these places. At the same time, it was possible to significantly reduce the waiting times for the mental health care of Mexican migrants.

In this study, as in others, the migrant population that visited the HW and MHU in 2021 reported higher mental health risks with symptoms related to depression, anxiety, psychosis and substance abuse (16–18, 24, 25). At the same time, women and youth have displayed the highest proportions of mental health risk (24). The most frequent symptoms or problems among women were related with depression, anxiety, suicidal ideation, psychosis, violence and sexuality, similarly to other research studies (26). Among men, the aforementioned symptoms were related to epilepsy, dementia, and substance use and abuse.

An analysis of the average age and age groups shows that these conditions are present in the younger population. According to an analysis by age group of the migrant population attended in both the HW and the MHU, the most common symptoms were related to depression, anxiety, suicidal ideation, and psychotic episodes. The studies by Rojas et al. (26) show emotional state and anxiety as mental illnesses prevalent among the childhood migrant population in Chile. The age group between 31 and 40 years of age most frequently showed symptoms related to violence and sexuality.

Another significant mental health aspect is substance abuse. According to some studies, the greater the exposure to North American culture, physical and emotional distance from partners or family, work pressures, fear of deportation, difficulty in expressing emotions, and social environment, the greater the increase in substance use (18, 27). In this study, substance use was reported as a mental health symptom or problem among 24% of the population, particularly among younger age groups.

It is important to note that although there were few people who agreed to be screened, the possibility of detecting mental health problems in one person, having the possibility of referring them to specialized care (psychological or psychiatrist) and that they agree to be treated, can translate into an improvement in the quality of life and those around them (family/community).

Based on the capacities deployed by the health promoters and the collaboration with the specialists of the UNAM (for the strengthening of the competencies for the evaluation, management and follow-up of mental health risks), during 2021, the strategy addressed conditions of stress, anxiety, major depression, psychological distress, risk of self-harm/suicide, use of psychoactive substances and post-traumatic stress; obtained in the screening to make remote specialized interventions available to them, based on scientific evidence, significantly reducing the wait time of those seeking care.

Evaluation, management and monitoring of mental health risks in community care was taught by the mental health promoters at the HW and MHU, in partnership with specialists from the Faculty of Psychology at UNAM. Mental health screening is also a community contact strategy, which makes it possible to detect the risk in early stages and interrupt the progression toward severity.

The strategy favored mental health risk screening and the collaborative strategy allowed the identification of true positives in specialized risk orders and true negatives in the community and primary level of care. This strategy also favored the reduction of the care gap at the community level, through the consent given to be contacted by specialists. Throughout the protocol, the link between the partners for mental health of the HW and MHU and the monitoring of remote psychological care increased.

## Limitations

The main limitation is the information source since we worked with secondary data and relied on information provided by those who attended both the HW and the MHU.

## Data availability statement

The datasets generated for this study are available on reasonable request to the corresponding author.

## Author contributions

ALJ: first draft, data analysis, and final draft. MR, SM, ALM, IL, and RC: revision and final draft. All authors contributed to the article and approved the submitted version.

## References

[1] Organización Mundial de la Salud (OMS). Plan de Acción sobre la Salud 1081 Mental 2013–2020. (2013). Available online at: https://apps.who.int/iris/bitstream/handle/10665/97488/9789243506029_spa.pdf (accessed May 24, 2022).

[2] Cuellar Rivas LX. La Salud Mental, un verdadero problema de salud pública. Rev Colomb Salud Libre. (2018) 13:5–8. 10.18041/1900-7841/rcslibre.2018v13n1.4985

[3] Mental Health Gap Action Programme (mhGAP). mhGAP Intervention Guide for Mental, Neurological and Substance Use Disorders in Non-Specialized Health Settings. Washington, D.C.: PAHO (2016). 27786430

[4] Morales CS. Impacto de la Pandemia por COVID-19 en la salud mental. Enferm Univ. (2021) 18:1–3. 10.22201/eneo.23958421e.2021.2.1218

[5] Organización Panamericana de la Salud. WHO—AIMS: Informe sobre los sistemas de salud mental en América Latina y el Caribe (2013).

[6] Organización Mundial de la Salud. Informe sobre el Sistema de Salud Mental en México IESM-OMS Instituto Nacional de Psiquiatría (2011).

[7] Barragán TL Flores MM Morales-Chaine S González VJ Martínez RMJ Ayala VH. Programa de Satisfactores Cotidianos. México: Secretaría de Salud (2014).

[8] Jurado D Mendieta-Marichal Y Martínez-Ortega JM Agrela M Ariza C Gutiérrez-Rojas L. Región mundial de origen y trastornos mentales comunes entre mujeres inmigrantes en España. J Immogr Minor Health. (2014) 16:1111–20. 10.1007/s10903-013-9927-0 24122225

[9] Bhugra D. Migration and mental health. Acta Psychiatr Scand. (2004) 109:243–58. 10.1046/j.0001-690X.2003.00246.x 15008797

[10] Odegaard O. Emigration and insanity: a study of mental disease among the Norwegian-born population of Minnesota. Acta Psych Scand. (1932) 7(Supplement 4):1–206.

[11] Bojorquez I. Salud mental y migración internacional Editorial. Colombia: Salud UIS, Universidad Industrial de Santander (2018).

[12] Vilar E Eibenschutz C. Migración y salud mental: Un problema emergente de salud pública. Rev Gerenc Polit Salud. (2007) 6:11–32.

[13] Achotegui J. Migración y salud mental. El síndrome del inmigrante con estrés crónico y múltiple (síndrome de Ulises). Gaceta Méd Bilbao. (2009) 106:163–71. 10.1016/S0304-4858(09)74665-7

[14] Cervantes R Castro F. Stress, coping and Mexican American mental health: a systematic review. Hisp J Behav Sci. (1985) 7:1–73. 10.1177/07399863850071001

[15] Murphy H. Migración, cultura y salud mental. Med Psicol. (1977) 7:677–84. 10.1017/S0033291700006334 594247

[16] Kirmayer LJ Narasiah L Munoz M Rashid M Ryder AG Guzder J. Common mental health problems in immigrants and refugees: general approach in primary care. CMAJ. (2011) 183:E959–67. 10.1503/cmaj.090292 20603342 PMC3168672

[17] Villaseñor S. Apuntes para una etnopsiquiatrí*a mexicana*. México: Universidad de Guadalajara, Centro Universitario de Ciencias de la Salud (2008), p. 13–29.

[18] Wallisch L Zemore SE Cherpitel CJ Borges G. Wanting and getting help for substance problems on both sides of the US-Mexico border. J Immigr Minor Health. (2016) 19:1174–85. 10.1007/s10903-016-0442-y 27286883 PMC5149114

[19] Bojorquez I Aguilera RM Ramírez J Cerecero D Mejía S. Common mental disorders at the time of deportation: a survey at the Mexico-United States Border. J Immigrant Minority Health. (2014) 16:1732–8. 10.1007/s10903-014-0083-y 25118675

[20] Rangel Gómez MG López Jaramillo AM Svarch A Tonda J Lara J Anderson EJ. Together for health: an initiative to access health services for the Hispanic/Mexican population living in the United States. Front Public Health. (2019) 7:273. 10.3389/fpubh.2019.00273 31608268 PMC6769102

[21] BD SICRESAL. Data Base Continuous Information System for Health Reporting (SICRESAL). (2021).

[22] Organización Internacional para las Migraciones (OIM). Informe sobre las migraciones en el mundo 2013: El bienestar de los migrantes y el desarrollo. Ginebra: OIM (2013).

[23] Tizón JL Salamero M Pellegro N San-José J Sáinz F Atxotegi J. Migraciones y salud mental: una revision empírica del tema desde una población asistencialmente delimitada. Psiquis. (1992) 13:169–87.

[24] Salaberría Irízar K de Corral P Sánchez A Larrea E. Características sociodemográficas, experiencias migratorias y salud mental en una unidad de apoyo psicológico a inmigrantes. Annu Clin Health Psychol. (2008) 4:5–14. 10.5944/rppc.vol.14.num.3.2009.4075

[25] Ruiz L Rodríguez D. Percepción de las necesidades en salud mental de población migrante venezolana en 13 departamentos de Colombia. Reflexiones y desafíos. Rev Gerenc Polit Salud. (2020) 19. 10.11144/Javeriana.rgps19.pnsm

[26] Rojas G Fritsch R Castro A Guajardo V Torres P Díaz B. Trastornos mentales comunes y uso de servicios de salud en población inmigrante. Rev Méd Chile. (2011) 139:1298–304. 10.4067/S0034-98872011001000008 22286729

[27] Torres TM. Vivencias de migrantes mexicanos sobre estados emocionales experimentados durante su proceso migratorio y el consumo de alcohol y drogas. Estud Front Mexicali. (2014) 15:247–70. 10.21670/ref.2014.29.a08

